# Ecologic and Sociodemographic Factors Associated with Seroprevalence of *Rickettsia* in Yucatan, Mexico

**DOI:** 10.3390/epidemiologia7020030

**Published:** 2026-02-25

**Authors:** Edgar Villarreal-Jimenez, Karla Dzul-Rosado, Fernando Puerto-Manzano, Jorge C. Guillermo-Herrera, Henry Pech-Noh, Nina Mendez-Dominguez

**Affiliations:** 1 Hospital Regional de Alta Especialidad de la Peninsula de Yucatan, Servicios de Salud del Instituto Mexicano del Seguro Social para el Bienestar IMSS-BIENESTAR, Merida 97130, Mexico; 2 Health Sciences, Universidad Autonoma de Yucatan, Merida 97203, Mexico; 3 Centro de Investigaciones Regionales Dr. Hideyo Noguchi, Universidad Autonoma de Yucatan, Merida 97203, Mexico

**Keywords:** seroepidemiologic studies, rickettsiosis, neglected diseases, environment

## Abstract

Background: Rickettsioses disproportionately affect vulnerable populations and are frequently misdiagnosed as other febrile illnesses in Yucatan, the Mexican state with the greatest diversity of *Rickettsia* spp. Although significant seroprevalence has been reported in rural communities, the last population-based study was conducted over two decades ago, despite environmental and social changes that have likely increased transmission risk. This study aimed to estimate the seroprevalence of spotted fever group (SFG) and typhus group (TG) of *Rickettsia* in an endemic area of southeastern Mexico. Methods: A cross-sectional analytical study was conducted among 390 participants. Indirect immunofluorescence was used to detect IgG antibodies against SFG and TG of *Rickettsia*. Sociodemographic characteristics of participants, along with environmental and community-level variables from their regions of residence, were analyzed. Results: The overall seroprevalence of both *Rickettsia* groups was 31.2%. Higher maximum temperatures were associated with an increase in *Rickettsia* seroprevalence (PR = 4.18; 95% CI: 3.40–5.14), while higher population density was associated with a decrease in seroprevalence (PR = 0.97; 95% CI: 0.96–0.98). Conclusions: *Rickettsia* seroprevalence in Yucatan remains high and is shaped by both environmental and demographic factors. These findings highlight the need to strengthen surveillance and prevention strategies that integrate ecological and social determinants within a One Health framework.

## 1. Introduction

Rickettsial diseases remain a significant yet underrecognized threat in tropical regions of Latin American—affecting vulnerable populations by causing serious morbidity and mortality [1]. In recent years, climate change has posed a challenge to public health, as it has been described as a potential factor involved in the spread, susceptibility, and increase in vector-borne diseases [2]. It is estimated that over 60% of emerging and re-emerging diseases belong to this group [3]. Among the most important vectors for disease transmission are arthropods, which exhibit strong adaptive capacity and can transmit more pathogens than other vectors [2,4,5]. Among the pathogens transmitted by these vectors are those of the *Rickettsia* genus, a group of obligate intracellular Gram-negative alphaproteobacteria [6], that are traditionally divided into four phylogenetic groups: (a) the ancestral group, (b) the transitional group (TRG), (c) the typhus group (TG), and (d) the spotted fever group (SFG) [7,8]. TG and SFG are of greatest medical relevance worldwide due to their broad distribution and potential severity [9]. In humans, rickettsioses cause a clinical picture characterized by headache, fever, rash, myalgia, or arthralgia. In severe cases, they may lead to pneumonitis, acute kidney injury, or even death.

Despite their similarities, important differences exist between the *Rickettsia* groups. TG rickettsiae are primarily transmitted by lice or fleas and often present with high fever but usually lack a prominent rash. In contrast, SFG rickettsiae are transmitted mainly by ticks and more commonly produce a macular or maculopapular rash; they are also associated with higher case-fatality rates compared with TG infections [9,10]. In endemic areas, timely diagnosis remains a challenge for physicians, as rickettsioses coexist with other febrile illnesses such as leptospirosis, dengue, chikungunya, and West Nile fever [11,12,13].

Over the past decade, outbreaks of rickettsioses have increased globally, with factors such as land use and human activities identified as potential contributors [12]. Previous studies have shown that ecological variables such as temperature, rainfall, and land-use patterns (deforestation, agriculture, and livestock) increase the likelihood of human infection [14,15,16,17]. Elevated temperatures accelerate vector metabolism, shorten feeding intervals, and promote active host-seeking [18]. Meanwhile, changes in land use increase the abundance and distribution of tick vectors, bringing humans, domestic animals, and wildlife into closer contact, intensifying transmission risk [17].

In Mexico, rickettsioses are considered neglected diseases, representing a risk for socially marginalized populations, particularly due to limited access to health services and the poor knowledge among physicians about these diseases [19], which hinders timely diagnosis. This especially concerns areas where other vector-borne diseases (VBDs) coexist, such as the state of Yucatan, where a high incidence of dengue, leptospirosis, and trypanosomiasis coincides with rickettsioses [20]; moreover, Yucatan state has the greatest variety of Rickettsial species in Mexico with the presence of TG (*R. typhi*), TRG (*R. felis*, *R. lusitaniae*), and SFG (*R. rickettsii*, *R. parkeri*, *Rickettsia* sp.) groups [21], and the presence of important vectors involved in transmission such as *Ixodes affinis*, *Rhipicephalus sanguineus*, *Amblyomma mixtum*, and *Ctenocephalides* spp. [21,22] has been documented. It has been described that the presence of dogs, cats, and opossums has been an important host in the cycle of the transmission of *Rickettsia* [23].

In previous serological studies conducted in rural communities with varying degrees of social deprivation in Yucatan, an IgG seropositivity to TG has been reported, especially *R. typhi,* ranging from 13% to 43%, and from 10% to 15% to *R. rickettsii* [24,25].

The Yucatan Peninsula has cross-demographic and socioeconomic shifts driven by industrial growth, tourism, and rapid urban development [26]. Changes in vegetation and habitat composition can alter microclimatic conditions and the distribution of wildlife hosts, creating environments that support higher tick survival and reproduction [27]. Over the last 20 years, Yucatan has experienced substantial landscape transformation driven by deforestation for agriculture, expansion of urban settlements, and the progressive fragmentation of remaining forest areas. Therefore, contact between feral and wild fauna and the local human population has become more frequent, potentially facilitating the circulation and transmission of VBD [28].

The objective of the present study was to identify the ecologic and sociodemographic factors associated with the seroprevalence of SFG and TG of *Rickettsia* in the southeastern state of Yucatan, Mexico.

## 2. Materials and Methods

### 2.1. Setting

**Figure 1:**
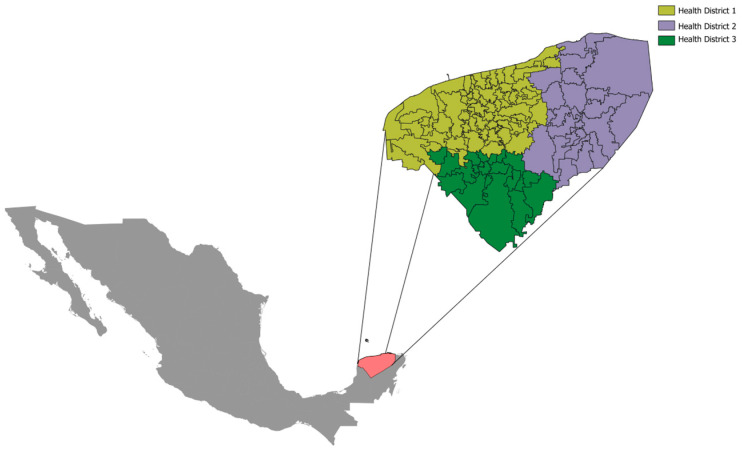
Distribution of health districts in Yucatan.

A cross-sectional study was conducted in the state of Yucatan, located in southeastern Mexico. It borders the Gulf of Mexico to the north, Quintana Roo to the east, Campeche and Quintana Roo to the south, and the Gulf of Mexico and Campeche to the west. The state was divided into health districts to identify ecological characteristics. These health districts are technical–administrative units that group municipalities with similar and specific epidemiological features. For epidemiological, sanitary, and administrative purposes, the state of Yucatan is subdivided into three health districts. Each district has a fixed number of municipalities: District 1 includes 60 municipalities, District 2 includes 24, and District 3 includes 21 (Figure 1).

### 2.2. Participants

We invited patients attending a tertiary-level hospital to participate in the study between January and March 2025. The hospital is located in the municipality of Merida, within Health District 1, and provides healthcare services to individuals without health insurance from all states. Inclusion criteria were being over 18 years of age and having no fever in the past three days at the sample collection. A probabilistic sample size was calculated to estimate prevalence in known populations, assuming an expected prevalence of 50%, a 95% confidence interval, and a 5% margin of error, resulting in a total sample size of a minimum of 384 participants. The sample for each region was determined using proportional allocation based on the population residing in each area, following the formula proposed by Wu and Thompson [29].

nh = n/N Nh

The proportional allocation method sets the sample size for a region (denoted nh) in proportion to the population size of that stratum (Nh). In the present study, we included 390 participants. Using a proportional allocation method, the sample was divided as follows: 298 participants from District 1, 58 from District 2, and 43 from District 3. All eligible participants consented to participate.

### 2.3. Serology

After providing informed consent, 5 mL of blood without anticoagulants was collected, and sociodemographic data were recorded. Sera were obtained by centrifugation at 1000 rpm in 10 min. Samples were frozen at ~80 °C until use. IgG antibody titers were determined by indirect immunofluorescence assay (IFA).

Human sera were examined via IFA protocol using crude antigens from *Rickettsia* (representing the SFG Rickettsiae) [30] and *Rickettsia typhi* (representing the TG *Rickettsiae*) [31] slides prepared in-house.

Cultured in Vero cells, and after an infection rate ≥ 70%, the cells were harvested and deposited in an antigen prepared according to standard. Dilutions of human serum were prepared in phosphate-buffered saline (PBS) containing 3% semi-skimmed milk (SSM), starting at 1:64 up to the endpoint dilution. Antigen-coated slides were blocked in PBS containing 3% SSM and 0.01% sodium azide; 10 μL of each dilution was placed into the wells, followed by incubation at 37 °C in a humid chamber for 45 min. The sheets were then washed with PBS containing Tween 20% to 0.1% for 10 min and then washed twice in the same solution for 10 min. Fluorescein isothiocyanate-conjugated goat anti-human IgG immune serum (ABCAM, Cambridge, UK) diluted 1:100 in PBS containing 3% SSM and 0.01% Tween20 was added to each well and incubated in a humid chamber at 37 °C for 40 min. Slides were washed once with PBS for 10 min, followed by staining with 5% Evans blue. The samples were observed under a fluorescence microscope at 400× magnification (Nikon Eclipse Ni-U, Tokio, Japan). A cutoff dilution of 1:128 was used to determine seropositivity, as proposed for endemic areas [32].

### 2.4. Sociodemographic and Ecological Variables

We included sociodemographic variables such as sex (male/female), age (years), age group, and health district of residence. For ecological variables, we included maximum temperature (°C), rainfall (mm), index of marginalization (high vs. not high), population density (persons/km^2^), agricultural area (percentage of land use), and altitude (meters). Each ecological variable was calculated for every district. All variables are described below:

- Population density (persons/km^2^): Number of inhabitants per km^2^ of each district.
- Altitude (m): Mean altitude above sea level, calculated in meters.
- Agricultural land (%): Percentage of land designated for annual or perennial crop cultivation.
- Average maximum temperature (°C): Average maximum temperature (°C) registered until 2024.
- Precipitation (mm): Average of precipitation records registered until 2024.
- Margination: Level of social deprivation, which includes limited access to education, inadequate housing, low income, and small locality size.

### 2.5. Statistical Analysis

Measures of central tendency and dispersion, as well as frequencies and percentages, were calculated, and two-group mean and proportion comparison tests were conducted, where a *p*-value < 0.05 was considered statistically significant. Ecologic factors were summarized using the median and interquartile range, and differences between health district clusters were evaluated using the Shapiro–Wilk test.

To assess the individual seropositivity analysis, sociodemographic variables were included as independent variables and the serological result was dichotomous (1 = positive, 0 = negative); results are reported in odds ratios (OR) for the unadjusted (UOR) and adjusted (AOR) model. Adjustment was performed for sex, age group, and health district.

To assess the association between overall seroprevalence (SFG and TG) and ecological factors at the health district cluster level, Poisson regression analysis was performed using cluster-wide ecological characteristics as independent variables and the reported prevalence ratio as a measure of association. All statistical analyses were performed using Stata^®^ version 14.

## 3. Results

### 3.1. Description of Sociodemographic Characteristics and Ecological Variables

A total of 390 participants were included, 58.9% (n = 230) were women, and the mean age was 53.03 ± 15.98 years. Cluster-level findings indicate that Health District 3 had the highest mean age (54.6 ± 16.6), while Health District 2 (53.6 ± 17.2) and Health District 1 (52.6 ± 15.6) followed. In the case of sex, women were the largest proportion in each district, representing more than half. Variables are further described in Supplementary Table S1.

The ecological variable displayed variability across the district. The Shapiro–Wilk test was used to assess whether each variable followed a normal distribution. Population, altitude, and precipitation showed notable departure from normality, suggesting skewed distributions driven by a few regions with particularly high values, as can be seen in Table 1.

### 3.2. Individual Factors Associated with Seropositivity of Rickettsia

An overall seroprevalence of 31.2% (122/390) for *Rickettsia* (SFG and TG) was found, with a seropositivity of 23.5% (92/390) to SFG, 3.33% (13/390) to TG, and a cross-reaction between the two groups was found in 4.5% (17/390). Seropositivity varied among health districts. Health District 3 had the largest proportion of positive results, with 44.18% (19/43). In the case of sociodemographic characteristics, the highest proportion of positive results was observed among women (58.2%). In addition, the mean age of seropositive individuals was higher (54.26 ± 15.59) than that of seronegative individuals. However, no significant differences in seropositivity were found between health districts, as shown in Table 2.

In the unadjusted logistic regression analysis, no significant associations were found between overall seropositivity to both SFG and TG and sociodemographic variables. Although, some age categories showed elevated odds ratios, but no statistically meaningful differences. After adjustment for potential confounders such as sex, age group, and health district, residing in a municipality belonging to Health District 3 was significantly associated with higher odds of seropositivity to *Rickettsia* (AOR = 2.03; 95% CI: 1.04–3.95; *p* = 0.037). In contrast, a marginal association was observed for the 25–44 age group (AOR = 4.53; 95% CI: 0.96–21.26).

The results suggest that geographic location, rather than individual demographic characteristics, plays an important role in exposure risk in the sample. The Hosmer–Lemeshow goodness-of-fit test indicated an adequate fit of the adjusted model (*p* = 0.508), supporting the validity of these findings (Table 3).

### 3.3. Ecologic Factors Associated with Seroprevalence

In the univariable Poisson regression, all ecologic characteristics showed a statistical association with *Rickettsia* seroprevalence (Table 4). Higher average maximum temperature demonstrated the strongest association with each 1 °C increase in seroprevalence (PR = 4.18; 95% CI: 3.40–5.14; *p* < 0.001). Also, the region with a high marginalization level showed higher seroprevalence, with a 61% increase compared with those of lower marginalization (PR = 1.61; 95% CI: 1.51–1.72; *p* < 0.001).

Conversely, higher population density was associated with a small but statistically significant decrease in seroprevalence (PR = 0.97; 95% CI: 0.96–0.98; *p* < 0.001), indicating a 3% reduction per unit increase in density. This suggests that the elevated seroprevalence observed in Health District 3 may be partly explained by the district’s lower population density.

## 4. Discussion

The overall seroprevalence of *Rickettsia* (SFG and TG) in this representative hospital-based sample from Yucatan was 31.2%, reflecting a higher level of exposure than that reported in early serological investigations, such as the 1999 population-based study documenting a prevalence of approximately 5% [33]. Such an increase aligns with patterns described in other settings where ecological transformation, new pathogen introductions, and changes in human–vector contact have altered local epidemiology [34]. In Yucatan, this shift may be partially explained by the recent detection of *R. parkeri*, an emerging SFG pathogen with increasing recognition in southeastern and northern Mexico [35,36,37]. Because *R. parkeri* infections tend to cause milder disease [36], undetected or subclinical infections could contribute to accumulating IgG seropositivity over time.

Another potential explanation relates to antibody transfer through blood donation, a mechanism previously suggested in endemic areas [38]. Although this study did not evaluate transfusion-related exposure, the tertiary hospital setting may capture individuals who have a history of blood donation. Future research assessing pathogen carriage in donors would help clarify this hypothesis.

Individual-level associations. Consistent with other Mexican studies [39], this analysis found no significant association between seropositivity and sex or age group, although seropositive individuals were slightly older on average, a pattern also described elsewhere [40]. Notably, the adjusted model identified residence in Health District 3 as a significant predictor of seropositivity (AOR = 2.03; 95% CI: 1.04–3.95), reinforcing the relevance of geographic context. This finding corresponds with the district’s ecological characteristics which have been linked to higher odds of rickettsial exposure in studies from Chile [41] and Colombia [42].

Geographic and ecological heterogeneity. Seroprevalence varied substantially across districts (29% to 44%), and this spatial heterogeneity mirrors international observations that vector distribution, land use, and climate conditions shape *Rickettsia* transmission [43,44]. The ecological analysis demonstrated that several environmental variables were significantly associated with seropositivity; higher maximum temperature showed the strongest association (PR = 4.18), consistent with evidence indicating that warm climates accelerate tick metabolism, shorten feeding intervals, and increase host-seeking behavior [15,16,45,46].

Marginalization was associated with a 61% increase in seroprevalence. Previous studies in rural Yucatan [24,25] and international data [16] have identified socioeconomic deprivation as a determinant of vector exposure due to inadequate housing, greater peridomiciliary vegetation, and increased outdoor labor. Higher altitude, agricultural surface, and precipitation also demonstrated significant associations, findings consistent with ecological studies linking vegetation structure, crop cultivation, and humidity to tick abundance and pathogen circulation [47,48].

In contrast, population density was inversely associated with seroprevalence (PR = 0.97). This pattern aligns with the literature from other regions, where more urbanized and densely populated areas exhibit reduced vector richness and diversity [49,50,51]. In Yucatan, peridomiciliary vegetation and backyard gardens in rural low-density communities have been associated with an increased risk of exposure [24,25].

Together, these results emphasize that seropositivity is not uniformly distributed but is shaped by the interplay between ecological variability and social context. This is consistent with the One Health perspective that integrates environmental change, land use, wildlife, and human behavior as intertwined determinants of transmission risk [19].

Broader regional context in Latin America. Our findings are consistent with serological patterns described across Latin America; in Chile, heterogeneous seroprevalence has been reported across climatic zones, correlating with temperature and vegetation [41]; in Colombia, multilevel analyses show district-level ecological features explaining a significant proportion of seropositivity variance, mirroring the geographic clustering observed in Yucatan [42].

Studies in Brazil and Argentina have likewise documented strong associations between land use, agricultural activity, and SFG prevalence [43,44]. These comparisons support the broader conclusion that environmental change, by deforestation, agricultural expansion, and urbanization among other conditions, alters the conditions that sustain *Rickettsia* vectors and hosts.

Public health implications. Given these findings, public health institutions have an opportunity to strengthen surveillance by incorporating rickettsial serology into routine monitoring, especially in high-temperature and high-marginalization areas. An improved diagnostic approach in primary care and rural services is also warranted. This should include:

(a) Rapid triage protocols for fever with rash or known vector exposure;
(b) Epidemiological risk assessment based on local ecological features;
(c) Empirical doxycycline initiation before laboratory confirmation;
(d) Community level prevention tailored to occupational and environmental risks.

Enhancing diagnostic capacity at regional laboratories and revising blood safety procedures may additionally contribute to early detection and reduction in transmission.

Study limitations. Our study limitations should be carefully considered. First, although proportional allocation was used, the sample is not strictly representative by district, and confidence intervals may be underestimated. Second, because recruitment occurred in a tertiary hospital, selection bias is possible, as patients may differ from the general community in health-seeking behavior or exposure patterns. This may lead to either overestimation or underestimation of true prevalence. Third, ecological analysis carries the inherent risk of ecological fallacy, meaning associations at the district level may not reflect individual-level exposure. Fourth, because of the relationship among the ecological variables, it was not possible to adjust the Poisson regression model; results were interpreted at the population level rather than at the individual level.

## 5. Conclusions

Almost one in every three adults in Yucatan show serological evidence of prior exposure to *Rickettsia*, underscoring that rickettsial infections remain a significant and underrecognized public health concern in the region. Seropositivity was not uniform across the state; instead, it varied according to the ecological and sociodemographic characteristics of each health district. Higher temperatures, greater marginalization, lower population density, and increased agricultural land use were strongly associated with elevated seroprevalence, highlighting that exposure risk is shaped by environmental and social determinants rather than individual characteristics alone.

These findings emphasize the need to strengthen surveillance and prevention strategies through a One Health framework that integrates ecological, climatic, and social indicators into early-warning systems. Improving timely clinical suspicion, particularly in high-risk rural and marginalized communities, together with enhanced diagnostic capacity and preventive outreach, could reduce delays in diagnosis and mitigate the risk of severe disease. Future research should incorporate molecular tools to clarify circulating species and employ community-based sampling to better estimate true population-level prevalence.

## Figures and Tables

**Table 1:** Ecological characteristics of health district clusters in Yucatan, Mexico.

	Median	Interquartile Range	25th Percentile	75th Percentile	Shapiro–Wilk W Test
Population density (persons/km^2^)	27.8	10.4	21.5	31.9	0.767
Altitude (m)	21.4	18.9	10.0	28.9	0.774
Agricultural land (%)	44.6	17.7	32.1	49.8	0.916
Average maximum temperature (°C)	33.0	0.30	32.9	33.2	0.999
Precipitation (mm)	958.9	329.3	834.7	1164.0	0.230

Values are presented as medians, interquartile ranges (IQR), and the 25th and 75th percentiles. The Shapiro–Wilk W test was used to assess departures from normality for each ecological variable.

**Table 2:** Comparison of sociodemographic characteristics and seropositivity for *Rickettsia* (SFG and TG).

	Sample n = 390 (%)	Seropositive n = 122 (%)	Seronegative n = 268 (%)	*p*-Value
Sex				
Male	160 (41.0)	51 (31.9)	109 (68.1)	0.833 *
Female	230 (59.0)	71 (30.9)	159 (69.1)	
Age (mean ± SD)	53.05 (±16.0)	54.26 (±15.6)	52.50 (±16.2)	0.314 ^+^
Age group				
18–24	17 (3.4)	2 (11.8)	15 (88.2)	0.311 ^+^
25–44	99 (25.4)	36 (36.4)	63 (63.6)	
45–49	50 (12.8)	13 (26.0)	37 (74.0)	
50–59	86 (21.8)	26 (30.2)	60 (69.8)	
60–64	28 (7.4)	7 (25.0)	21 (75.0)	
>65	110 (28.2)	38 (34.5)	72 (65 5)	
Health districts				
1	289 (74.1)	85 (29.4)	204 (70.6)	0.149 ^+^
2	58 (14.9)	18 (31.0)	40 (69.0)	
3	43 (11.0)	19 (44.2)	24 (55.8)	

* Two-group mean comparison test; ^+^ X2 two-group proportion test; SD: Standard deviation.

**Table 3:** Associations between sociodemographic characteristics and seropositivity of *Rickettsia* (SFG and TG).

	UOR *	95% Confidence Interval		*p*-Value	AOR +	95% Confidence Interval		*p*-Value
		Lower	Upper			Lower	Upper	
Sex (Men)	1.0	0.7	1.6	0.833	1.1	0.7	1.7	0.710
Age group								
18–24	Reference value				Reference value			
25–44	4.3	0.9	19.9	0.062	4.5	0.9	21.3	0.055
45–49	2.6	0.5	13.1	0.237	2.7	0.5	13.8	0.223
50–59	3.3	0.7	15.2	0.149	3.4	0.7	16.0	0.126
60–65	2.5	0.4	13.7	0.222	2.4	0.4	13.5	0.316
>65	3.9	0.8	18.2	0.077	4.1	0.8	19.0	0.072
Health district								
1	Reference value				Reference value			
2	1.1	0.6	2.0	0.805	1.1	0.6	2.0	0.834
3	1.1	1.0	3.6	0.054	2.0	1.0	3.9	0.037

* Unadjusted; + adjusted odds ratio.

**Table 4:** Univariate Poisson regression associations between seroprevalence to *Rickettsia* (SFG and TG) and ecologic characteristics in Yucatan.

Ecologic Characteristics	PR	95% Confidence Interval		*p*-Value
		Lower	Upper	
Population density (person/km^2)^	0.97	0.96	0.98	<0.001
Altitude (m)	1.03	1.03	1.04	<0.001
Agricultural land (%)	1.02	1.01	1.02	<0.001
Average maximum temperature (°C)	4.18	3.40	5.14	<0.001
Precipitation (mm)	1.00	1.00	1.00	<0.001
Marginalization level (High)	1.61	1.51	1.72	<0.001

PR: Prevalence ratio.

## Data Availability

Data is available at Researchgate linked to authors profiles, https://doi.org/10.13140/RG.2.2.20311.53920 (accessed on 9 February 2026).

## Supplementary Materials

The following supporting information can be downloaded at: https://www.mdpi.com/article/10.3390/epidemiologia7020030/s1, Table S1. Description of Variables Included in the Sociodemographic and Ecologic Analysis.

## Abbreviations

SFG	Spotted fever group
TG	Typhus group
VBDs	Vector-borne diseases
IFA	Indirect immunofluorescence assay
OR	Odds ratios
