# Supplementary material for: Ecologic and Sociodemographic Factors Associated with Seroprevalence of Rickettsia in Yucatan, Mexico
Source: Epidemiologia (Basel). 2026 Feb 25;7(2):30. doi: 10.3390/epidemiologia7020030 (PMC13010737; doi:10.3390/epidemiologia7020030)
Supplement: Supplementary file 1 [file epidemiologia-07-00030-s001.zip › epidemiologia-3953435-supplementary.pdf]

Table S1. Description of Variables Included in the Sociodemographic and Ecologic

	Variable	Description
Sociodemographic	Sex	Classified as male or female.
	Age (years)	Age of the individual in completed years.
	Age group	Categorical grouping of age (18-24; 25-44; 45-49; 50-59; 60-64; >65)
	Health district	Administrative health district where the individual resides.
Ecologic	Population density (persons/km²)	Number of inhabitants per square kilometer in each district
	Altitude (meters)<br>Altitude (meters)	Mean altitude above sea level for each district, measured in meters.
	Agricultural Land use (%)	Percentage of district land dedicated to annual or perennial crop cultivation.
	Average maximum temperature (°C)	Average maximum temperature recorded in each district up to 2024.
	Precipitation (mm)	Average precipitation recorded in each district up to 2024
	Marginalization index	Level of social deprivation; categorized as high vs. not high. Includes factors such as educational access, housing conditions, income, and locality size.

Analysis
